# Seasonal impact of grazing, viral mortality, resource availability and light on the group-specific growth rates of coastal Mediterranean bacterioplankton

**DOI:** 10.1038/s41598-020-76590-5

**Published:** 2020-11-13

**Authors:** Olga Sánchez, Isabel Ferrera, Isabel Mabrito, Carlota R. Gazulla, Marta Sebastián, Adrià Auladell, Carolina Marín-Vindas, Clara Cardelús, Isabel Sanz-Sáez, Massimo C. Pernice, Cèlia Marrasé, M. Montserrat Sala, Josep M. Gasol

**Affiliations:** 1 Departament de Genètica i Microbiologia, Universitat Autònoma de Barcelona, 08193 Bellaterra, Catalunya Spain; 2 Centro Oceanográfico de Málaga, Instituto Español de Oceanografía, 29640 Fuengirola, Málaga, Spain; 3 Departament de Biologia Marina i Oceanografia, Institut de Ciències del Mar, ICM-CSIC, 08003 Barcelona, Catalunya Spain; 4 Instituto de Oceanografía y Cambio Global (IOCAG), Universidad de Las Palmas de Gran Canaria (ULPGC), Telde, 35214 Spain; 5 Escuela de Ciencias Biológicas, Universidad Nacional, Heredia, 40101 Costa Rica

**Keywords:** Microbiology, Ocean sciences

## Abstract

Estimation of prokaryotic growth rates is critical to understand the ecological role and contribution of different microbes to marine biogeochemical cycles. However, there is a general lack of knowledge on what factors control the growth rates of different prokaryotic groups and how these vary between sites and along seasons at a given site. We carried out several manipulation experiments during the four astronomical seasons in the coastal NW Mediterranean in order to evaluate the impact of grazing, viral mortality, resource competition and light on the growth and loss rates of prokaryotes. Gross and net growth rates of different bacterioplankton groups targeted by group-specific CARD-FISH probes and infrared microscopy (for aerobic anoxygenic phototrophs, AAP), were calculated from changes in cell abundances. Maximal group-specific growth rates were achieved when both predation pressure and nutrient limitation were experimentally minimized, while only a minimal effect of viral pressure on growth rates was observed; nevertheless, the response to predation removal was more remarkable in winter, when the bacterial community was not subjected to nutrient limitation. Although all groups showed increases in their growth rates when resource competition as well as grazers and viral pressure were reduced, *Alteromonadaceae* consistently presented the highest rates in all seasons. The response to light availability was generally weaker than that to the other factors, but it was variable between seasons. In summer and spring, the growth rates of AAP were stimulated by light whereas the growth of the SAR11 clade (likely containing proteorhodopsin) was enhanced by light in all seasons. Overall, our results set thresholds on bacterioplankton group-specific growth and mortality rates and contribute to estimate the seasonally changing contribution of various bacterioplankton groups to the function of microbial communities. Our results also indicate that the least abundant groups display the highest growth rates, contributing to the recycling of organic matter to a much greater extent than what their abundances alone would predict.

## Introduction

Growth rates, along with the rates of mortality, determine the biomass levels of bulk bacterioplankton communities and of specific taxonomic groups, and set the contribution of microorganisms to ocean biogeochemical cycles (see review by Kirchman^1^). Their determination is thus crucial to understand which members of the bacterioplankton contribute to the flow of elements and energy to higher trophic levels. Overall, bulk bacterial growth rates are low, ranging between 0.05 and 0.10 day^−1^ in oligotrophic marine regions (1 division every one or two weeks)^2^, but gross growth rates of particular groups can be much higher, with generation times in the order of a few days or hours^1,3–5^.

Different biotic and abiotic factors can modulate net growth rates, since prokaryotes are largely limited by resource availability (bottom-up control), and grazing by protists and viral lysis (top-down control) constitute the main sources of mortality^4,6–9^. In temperate marine ecosystems, temperature together with light largely shape the environmental conditions and thus determine the seasonal changes in prokaryote abundance and species composition^10–14^. Actually, the importance of temperature as a regulator of growth rate has been documented at a seasonal scale^15–17^. However, the effects of sunlight on bacterioplankton are diverse and complex, and it is extremely challenging to predict community responses to light changes, since these responses may be dependent on light quality and intensity, but also on nutrient availability, previous light exposure or water column vertical mixing, in addition to community composition^18–23^. Thus, the role that light plays on the growth rate of different taxa under natural conditions is far from being clear.

It is well known that marine prokaryotic and protist communities show marked and reoccurring seasonal patterns^13,14,24–29^, and it appears from some temporal studies that marine viruses also display seasonal dynamics^30–32^. Correspondingly, seasonal variations of grazers and virus-mediated mortality have been documented^33^. Likewise, variability of nutrient concentrations and bacterioplankton nutrient limitation along seasons has also been described^34–38^. Despite this wealth of studies, information about seasonal changes in the bottom-up and top-down controls on bacterial growth rates is scarce. Some previous studies have put the focus on the importance of these factors in determining the net and gross growth rates of different bacterioplankton groups^4,5,9,39,40^, but most of these studies were restricted to specific short time periods, and, as far as we know, only two of them looked at the factors controlling bacterial growth rates along longer time scales in a coastal upwelling system^3^ or an estuary^39^, but none in an oligotrophic system. Furthermore, the above-mentioned studies did not test the effect of light as modulator of growth. Thus, the seasonal interplay of temperature and light effects on bacterial growth has barely been taken into consideration.

To evaluate the impact of top-down (protists and viruses) and bottom-up (resources) controls on bacterial growth rates under different light conditions, we conducted manipulation experiments during the four astronomical seasons and determined the net and gross growth rates of different CARD-FISH-determined bacterioplankton groups at the Blanes Bay Microbial Observatory (BBMO), an oligotrophic coastal site in the Northwest Mediterranean. Additionally, the community of aerobic anoxygenic phototrophs (AAP), particularly relevant for their assumed responses to light and fast growth rates^4^, was also examined. Growth rates were calculated from changes in cell numbers over time for major phylogenetic bacterioplankton groups and AAP. Furthermore, mortality rates were estimated from the difference between the gross and the net growth rates. The results contribute to our knowledge of the magnitude of marine prokaryotic growth rates and the role that top-down and bottom-up pressures, as well as light, play in controlling them over a seasonal cycle.

## Methods

### Sample collection and environmental data

Seawater samples were collected from the Blanes Bay Microbial Observatory (BBMO), a shallow coastal site located 1 km offshore on the Mediterranean coast (41°40′N, 2°48′E), approximately 70 km north of Barcelona, and from which we have plenty of previous information (e.g. Gasol et al.^41,42^). Four experiments were conducted with surface water collected on 21 February 2017, 26 April 2017, 5 July 2017 and 7 November 2017, respectively for the Winter, Spring, Summer and Fall experiments. Seawater was sieved through a 200-µm mesh and transported to the laboratory within 2 h. Water temperature and salinity were measured in situ with a CTD (conductivity, temperature, and depth) SAIV SD204 probe, photosynthetically active radiation (PAR) at the sampling site was measured with a multichannel filter radiometer (PUV-2500; Biospherical Instruments Inc.), and light penetration was estimated using a Secchi disk. The concentration of inorganic nutrients was determined spectrophotometrically with an Alliance Evolution II autoanalyzer according to standard procedures^43^. Chlorophyll *a* (Chl *a*) concentration was measured from acetone extracts by fluorometry. Abundances of heterotrophic bacteria, photosynthetic phytoplankton and viruses were measured by flow cytometry with a FACSCalibur (BectonDickinson) flow cytometer^44^, and discrimination of populations with high nucleic acid content (%HNA) was done as described previously^44^. Heterotrophic nanoflagellates (HNF) were filtered onto polycarbonate 0.6-µm filters and stained with 4′, 6-diamidino-2-phenylindole (DAPI, final concentration 1 µg·mL^−1^)^45^ and counted in an Olympus BX61 epifluorescence microscope. Bacterial biomass production was estimated measuring the incorporation of leucine, after adding 40 nM [^3^H]leucine^46^, with the modifications described by Smith & Azam^47^. Incorporation was converted to biomass production using a conversion factor of 1.5 kgC mol Leucine^−1^, close to the seasonal average for Blanes Bay^48^.

### Experimental setup

At each season, seawater was exposed to six experimental treatments: (1) whole unfiltered seawater, both in light/dark cycles and in continuous dark (control light [CT_L] and control dark [CT_D] respectively), (2) seawater prefiltered with a 1-µm filter to remove large predators while keeping most bacteria, both in light/dark cycles and in continuous dark (predator-reduced light [PR_L] and predator-reduced dark [PR_D] respectively), (3) a 1:4 dilution of whole seawater with 0.2-µm-filtered seawater to reduce both predation and competition for nutrient and carbon resources among bacteria (diluted treatment in light/dark cycles [DI_L]), and (4) a 1:4 dilution of whole seawater with seawater filtered through a 30-kDa VivaFlow cartridge to reduce predation, viruses and resource competition (virus-reduced treatment in light/dark cycles [VR_L]). The samples were subjected to these manipulations, that lasted ca. 20 h from sampling to start of the experiment, and were then distributed into 9-L Nalgene bottles that were incubated in triplicate for 1.5–2 days in a large water bath (200 L) with circulating seawater to maintain the temperature close to in situ conditions. The light treatments were limited to PAR by maintaining the bottle incubations under natural light conditions with the exclusion of UV radiation, using two layers of an Ultraphan URUV Farblos Filter and a net that reduced light intensity to roughly mimic the light conditions of a water depth of 3 m, calculated from the transparency measured in situ at the sampling time. PAR radiation was monitored continuously with a radiometer placed inside the incubation water bath and with the same covers. For dark treatments, the bottles were completely covered with black plastic to prevent light exposure. Samples were collected regularly for measurements of leucine incorporation, HNA content, viruses and inorganic nutrients as described above, as well as for the enumeration of aerobic anoxygenic phototrophs (AAP), and determination of catalyzed reporter deposition fluorescence in situ hybridization (CARD-FISH).

### Enumeration of AAP by epifluorescence microscopy

In each experiment and replicated bottle, 2 subsamples distributed in time along 1.5–2 days were collected from those treatments which combined light and dark bottles (control and predator-reduced treatments), fixed with 2% formaldehyde, and filtered onto a 0.2-µm polycarbonate filter. Cells were stained with DAPI (final concentration 1 µg·mL^−1^) and counted by using an Olympus BX61 epifluorescence microscope as described previously^49^. Briefly, three fluorescence images were captured for each frame. First, total DAPI-stained bacteria were recorded in the blue part of the spectrum, Chl *a* autofluorescence was subsequently recorded in the red part of the spectrum, and finally, both BChl *a* and Chl *a*-containing organisms were recorded in the infrared part of the spectrum (> 850 nm). For each sample, at least 10 frames were recorded and analyzed semimanually using AnalySiS software (Soft Imaging Systems) to distinguish between heterotrophic bacteria, picocyanobacteria, and AAP. AAP counts were finally obtained by subtracting the contribution of Chl *a*-containing organisms to the infrared image.

### CARD-FISH and calculation of specific growth rates

For bacterial abundance determination, 4 subsamples distributed in time along 1.5–2 days were collected from each triplicated bottle, fixed with 2% paraformaldehyde, and filtered onto a 0.2-µm polycarbonate filter. CARD-FISH was performed as described by Pernthaler et al.^50^ using the following probes: a mixture of EUB338-I, -II, and -III for Eubacteria^51,52^, Ros537^53^ for *Rhodobacteraceae*, SAR11-411R^54^ for SAR11, Gam42a^51^ for Gammaproteobacteria, Alt1413^53^ for *Alteromonadaceae*, NOR5-730^53^ for NOR5/OM60, and CF319a^51^ for Bacteroidetes. Counterstaining of CARD-FISH preparations was done with DAPI (final concentration 1 µg mL^−1^). DAPI and CARD-FISH-stained cells were counted by fully automated microscopy^55,56^ with a Zeiss Axio Imager.Z2M using the automated image analysis software ACME Tool^57^. Growth rates were calculated using the time course measurements of the absolute abundance of each phylogenetic group (cells mL^−1^), and derived from the slope of the regression between the ln of abundance versus time, for the time interval during which exponential growth was observed.

### Determination of mortality rates

To calculate mortality rates, as well as the constraints on growth rates derived from competition for resources, we used the specific growth rates obtained from treatments under light/dark cycles, since dark conditions were only carried out for CT and PR treatments and did not allow to estimate mortality rates. The following equations modified from Evans et al.^58^ and Pasulka et al.^59^ were utilized:

$$\begin{aligned} & K_{CT} = \mu - \left( {m_{g} + \, r_{c} + \, m_{v} } \right) \\ & k_{PR} = \mu - \left( {r_{c} + \, m_{v} } \right) \\ & k_{DI} = \mu - \left( {D\cdot m_{g} + \, D\cdot r_{c} + \, m_{v} } \right) \\ & k_{VR} = \mu - D\cdot\left( {m_{g} + \, r_{c} + \, m_{v} } \right) \\ \end{aligned}$$

where *k* is the measured net growth rate for each treatment (CT: control, PR, predator-reduced, DI: diluted, VR: virus-reduced), *µ* the gross growth rate, D the dilution factor (in this study, 0.25), *m*_*g*_ the mortality due to grazers, *r*_*c*_ the losses due to resource limitation and *m*_*v*_ the mortality due to viruses. Factor *r*_*c*_ was not included in the equations from Evans et al^58^. and Pasulka et al^59^, but it was considered here in order to account for bottom-up constraints on growth rate due to resource limitation.

### Statistical analysis

All statistical analyses were conducted in R (version 3.6.2)^60^. Analysis of variance was done to test for differences in growth and mortality rates depending on experiment, bacterioplankton group or treatment with Tukey HSD post hoc comparisons at the 5% significance level. Differences in the growth response among treatments and experiments were visualized using hierarchical clustering (Ward's method) and cluster uncertainty was tested with the *pvclust* package v2.2 (999 permutations)^61^. Permutational tests (PERMANOVA) were employed to examine the differences among seasons, treatments and sampling times.

## Results

### Initial environmental parameters and bacterial community structure

The in situ physicochemical and biological parameters were quite different in the various seasons (Table 1). Chl *a* and inorganic nutrient concentrations were higher in winter, with the exception of ammonium, which was higher in spring. Picoeukaryotic phytoplankton abundance was also higher in winter while *Synechococcus* abundance was so in spring and fall. Bacterial production, nonetheless, reached the highest value in summer, although prokaryotic abundance in this period was low (in the order of 10^5^ cells mL^−1^) compared to the rest of seasons (in the order of 10^6^ cells mL^−1^). In contrast, the abundances of heterotrophic nanoflagellates were fairly similar among seasons, while the viral abundance was particularly low in spring and reached the highest value in fall.

**Table 1:** Physicochemical and biological parameters of the initial samples in the different experiments.

Variable	Winter	Spring	Summer	Fall
Date	2017/2/20	2017/4/25	2017/7/4	2017/11/6
Temperature (°C)	12.8	14.8	23.1	19.5
Salinity	38.01	38.06	38.02	37.70
Secchi disk depth (m)	8	20	20	19
Surface PAR (µmol photons m^−2^ s^−1^)	546	569	789	224
Chlorophyll *a* (µg L^−1^)	1.20	0.43	0.13	0.46
[PO_4_^3−^] (µM)	0.044	0.028	0.015	0.025
[NH_4_^+^] (µM)	0.214	1.567	0.431	0.200
[NO_2_^−^] (µM)	0.280	0.119	0.036	0.040
[NO_3_^−^] (µM)	1.167	0.357	0.034	0.155
[SiO_4_^4−^] (µM)	1.507	1.194	0.690	0.663
DOC (µM)	63.8	65.7	86.2	77.9
Prokaryotic abundance (cells mL^−1^)	1.04 × 10^6^	1.01 × 10^6^	7.28 × 10^5^	1.58 × 10^6^
Bacterial production (µgC L^−1^ day^−1^)	2.57	3.03	4.62	1.34
Leu-based prokaryotic specific growth rate (day^−1^)	0.033	0.047	0.139	0.032
% HNA prokaryotic cells	61.6	48.0	46.6	26.9
Heterotrophic nanoflagellate abundance (cells mL^−1^)	1.24 × 10^3^	1.65 × 10^3^	1.49 × 10^3^	1.03 × 10^3^
*Synechococcus* abundance (cells mL^−1^)	1.06 × 10^4^	4.43 × 10^4^	1.70 × 10^4^	3.45 × 10^4^
Picoeukaryote abundance (cells mL^−1^)	1.61 × 10^4^	6.44 × 10^3^	1.27 × 10^3^	2.38 × 10^3^
Viral abundance (viruses mL^−1^)	9.89 × 10^6^	1.16 × 10^6^	7.75 × 10^6^	1.90 × 10^7^

The in situ average contributions of various CARD-FISH-determined groups to total prokaryotic abundance in this study are presented in Table 2. In general, SAR11 dominated in all samples and seasons, followed by Bacteroidetes, while proteobacterial groups such as *Rhodobacteraceae*, or the NOR5/OM60 clade and the family *Alteromonadaceae*, both belonging to Gammaproteobacteria, were found in lower abundances. Overall, despite a particular exception (Eubacteria in summer had a very low contribution to DAPI values), their abundance was quite regular among seasons and followed the same trend as that reported in previous studies carried out in the same site (see average in Table 2).

**Table 2:** In situ average contribution to total bacterial abundance ± standard deviation of the different bacterioplankton groups represented as percentages of DAPI-positive cells in the different experiments of this study (2017) and in other years at the BBMO.

Group	Winter (Feb 2017)	Spring (April 2017)	Summer (July 2017)	Fall (Nov 2017)	Average^a^
Eubacteria	73.7	76.8 ± 2.2	52.1 ± 2.2	73.3	74.1 ± 10.2
Bacteroidetes	15.9	12.9 ± 1.0	8.4 ± 1.1	11.9	11.6 ± 3.0
*Rhodobacteraceae*	4.5	3.1 ± 1.4	3.0 ± 0.1	2.4	3.6 ± 1.9
SAR11	43.5	38.9 ± 1.6	35.0 ± 6.7	45.1	27.7 ± 14.1
Gammaproteobacteria	2.0	3.2 ± 0.6	3.3 ± 1.3	5.8	7.7 ± 11.0
*Alteromonadaceae*	1.9	0.1 ± 0.0	2.6 ± 0.4	4.3	2.0 ± 1.6
NOR5/OM60	1.2	0.7 ± 0.4	2.0 ± 0.5	1.7	2.4 ± 1.5
AAP	8.9	4.9 ± 1.8	16.7 ± 5.0	10.4	6.0 ± 1.4

Bacterioplankton groups were detected with specific HRP-probes Eub 338-I, -II, -III (Eubacteria), CF319a (Bacteroidetes), Ros537 (*Rhodobacteraceae*), SAR11-441R (SAR11 clade), Gam42a (Gammaproteobacteria), Alt1413 (*Alteromonadaceae*) and NOR5-730 (NOR5/OM60 cluster). AAP: aerobic anoxygenic phototrophs, determined by infrarred microscopy.

^a^Data from Ferrera et al^4^, Sánchez et al^5^, and Alonso-Sáez et al^10^.

### Effect of bottom-up and top-down controls on bacterial heterotrophic production and the percentage of HNA cells

Bacterial heterotrophic production (measured as leucine incorporation rates) exhibited large differences among seasons and the effect of the different manipulations was likewise variable (Supplementary Fig. S1). Overall, the responses of bacterial production pointed to predation as the main mechanism of control of bacterial growth in winter, and to a lesser extent in spring and fall, while in summer predation seemed to be less important.

Similarly, bacteria with high nucleic acid content (HNA), which can be used as a single cell-based proxy of global population activity^62,63^ and/or can represent the proportion of copiotrophic cells^64^, showed differences between seasons (Supplementary Fig. S1). In winter, HNA values reached the highest percentages determined among seasons (ranging between 83 and 85%), when bottom-up and top-down controls were reduced. Likewise, in summer, HNA cells achieved similar values in the DI_L and VR_L treatments (around 80%). In contrast, in spring and fall the % of HNA cells did not reach the high values observed in winter or summer upon the experimental manipulations.

### Effect of bottom-up and top-down controls on prokaryotic abundances

There was a general rise in total prokaryotic numbers (as estimated from DAPI counts) during the length of the incubations (36 h in winter and summer and 48 h in spring and fall), which was more pronounced after the manipulation of top-down and bottom-up factors (Supplementary Fig. S2 and Table S1). On the other hand, the magnitude of the increase of group-specific bacterioplankton abundances largely depended on the group examined (Supplementary Table S1). For all seasons, the *Alteromonadaceae* and consequently the Gammaproteobacteria, underwent the highest increment in abundance after relieving the bottom-up and top-down controls. In winter, spring and fall, this group achieved an important rise in abundance in all treatments but the controls, while in summer this increase was remarkable only when resource competition and viral pressure were reduced (DI_L and VR_L treatments).

**Figure 1:**
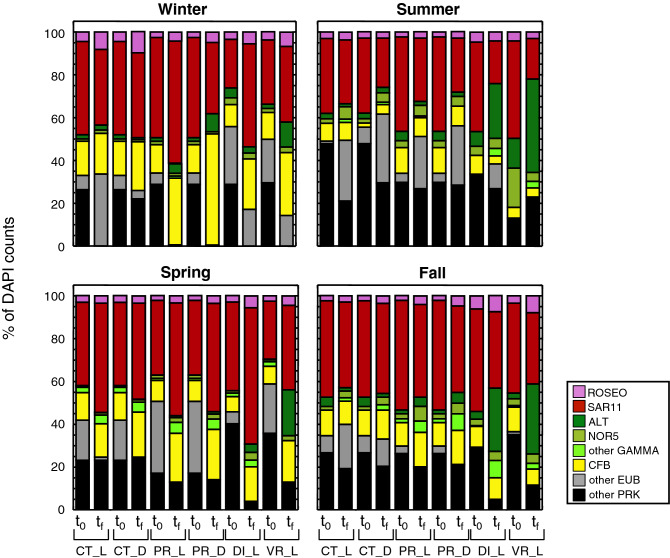
Stack columns showing the mean abundance of the different phylogenetic groups as percentage of total DAPI counts for each season at the beginning (t_0_) and at the end (t_f_) of the different treatments (36 h in winter and summer and 48 h in spring and fall). CT_L, control under PAR; CT_D, control in the dark; PR_L, predator-reduced treatment under PAR; PR_D, predator-reduced treatment in the dark; DI_L, diluted treatment under PAR; VR_L, virus-reduced treatment under PAR. ROSEO, *Rhodobacteraceae*; SAR11, SAR11 clade; ALT, *Alteromonadaceae*; NOR5, NOR5/OM60 clade; GAMMA, Gammaproteobacteria; CFB, Bacteroidetes; EUB, Eubacteria; PRK, prokaryotes.

Figure 1 shows the percentage of the relative abundance of each specific group of prokaryotes at the beginning and at the end of every experiment and treatment. Remarkably, although all groups increased in cell numbers after manipulations, SAR11 was in most cases the group which still dominated the prokaryotic community, with the exception of summer and fall, where *Alteromonodaceae* reached the highest relative abundance in the DI_L and VR_L treatments. Also in winter in the PR_D treatment, Bacteroidetes achieved higher relative abundance values than SAR11. Interestingly, the percentage of microorganisms considered as “other prokaryotes”, that is, the portion not detected with any of the used probes, decreased at the end of almost all manipulation experiments, suggesting that a certain fraction of prokaryotes could initially be inactive or dormant, or alternatively it could be an indication of false negatives, not targeted by the used probes. This fraction, reaching values of up to 48% in summer at t_0_, is likely to belong mainly to Bacteria, since the reported fraction of Archaea in previous studies at the BBMO was much lower than that of Bacteria, being at most up to 9% of DAPI counts^10^.

To visualize differences in the growth response among manipulation treatments and seasons, we used hierarchical clustering based on the cell abundances of the targeted groups. Within experiments, we observed that in general dilution and virus-reduced treatments clustered together, clearly differentiating from the predator-reduced and the control treatments. Differences between predator-reduced and control treatments were variable among seasons as seen by the clustering in each experiment (Supplementary Fig. S3). Additionally, sampling time also influenced sample clustering. When comparing all treatments and experiments (Supplementary Fig. S3), the grouping of dilution and virus-reduced treatments was maintained but samples further tended to cluster according to season (winter and spring experiments on one side, and summer and fall samples on the other). Permutation tests on the whole dataset confirmed that 59% of the observed variance could be explained by the three variables tested, with season and treatment explaining roughly the same amount of variation and sampling time having less explanatory power (see Supplementary Table S2).

### Effect of bottom-up and top-down controls on group-specific growth rates

**Figure 2:**
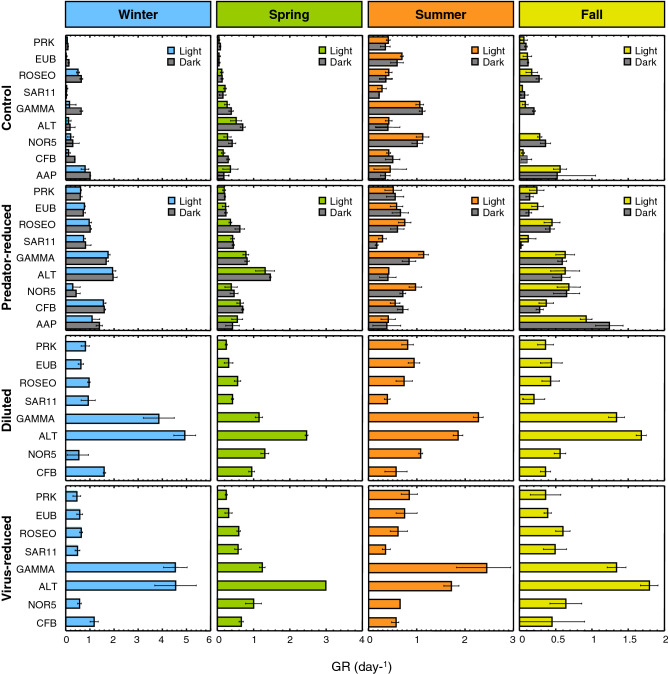
Mean growth rates of the different phylogenetic groups for each season in the control, predator-reduced, diluted and virus-reduced treatments. Error bars represent the standard deviations for three replicated incubations. PRK, total prokaryotes; EUB, Eubacteria; ROSEO, *Rhodobacteraceae*; SAR11, SAR11 clade; GAMMA, Gammaproteobacteria; ALT, *Alteromonadaceae*; NOR5, NOR5/OM60 clade; CFB, Bacteroidetes; AAP, aerobic anoxygenic phototrophs.

Time-course measurements of the absolute abundance of each phylogenetic group (i.e., in cells mL^−1^) were used to determine the net growth rates (in the control treatments) and close-to-gross growth rates (i.e. the rates upon reduction in grazing, viral and resource availability pressure) for the different prokaryotic groups in each season (Fig. 2). The total prokaryotic community (as estimated from DAPI counts) grew at about the same rate as the whole bacterial community (determined by CARDFISH with the Eubacterial probe) for each season and in all treatments, and presented higher values in summer (in concordance with the specific activity rates measured at the start of the experiment, based on leucine incorporation, Table 1). In general, the manipulation treatments resulted in the increase in the growth rate of all groups studied, being maximal in the diluted and virus-reduced treatments. The gammaproteobacterial family *Alteromonadaceae* (and consequently the Gammaproteobacteria) was the group with the highest gross growth rates in all seasons in DI_L and VR_L treatments. The most abundant group, SAR11, showed maximal net growth rates in spring and summer, but the maximal net growth rates in the control treatments corresponded to the Gammaproteobacteria in summer. Table 3 displays a summary of the values of minimal and maximal growth rates at the BBMO for all groups investigated in this work and in other studies from the same location. Overall, the values found in this study are within the range of those previously reported (Table 3). *Alteromonadaceae* had systematically the highest growth rates, achieving a value of 4.9 day^−1^ in winter, slightly lower than some values previously reported for this group (5.8 day^−1^, Ferrera et al.^4^, Table 3). In contrast, SAR11 displayed always the lowest growth rates (between 0.03 and 1.8 day^−1^).

**Table 3:** Summary of minimal and maximal growth rates (day^−1^) for the different bacterioplankton groups measured in this work (year 2017) and in other studies at the BBMO.

Date	PRK	Eub338-I, -II, -III	Ros537	SAR11	Gam42a	Alt1413	NOR5-730	CF319a	AAP
June 09^a^	0.7–**1.7**	0.7–**1.7**	0.9–**2.9**	0.1–**1.8**	1.0–3.6	2.3–5.4	1.7–2.8	0.7–1.5	1.6–3.7
July 09^a^	0.2–1.3	0.2–1.3	0.3–1.9	0.1–1.5	1.0–3.4	1.4–**5.8**	1.3–**2.9**	0.5–1.6	0.3–2.4
May 10^b,c^	0.3–**1.7**	0.2–1.5	0.5–3.7	0.5–1.5	1.0–2.7	1.0–4.7	0.6–2.3	0.2–**2.2**	1.2–**5.9**
July 11^b,c^	0.2–0.6	0.2–0.7	0.4–1.8	0.1–0.5	0.5–1.5	0.4–3.4	**0.2**–1.3	0.2–0.6	0.5–2.2
February 17	**0.1**–0.8	**0.0**–0.8	0.5–1.0	**0.0**–0.9	**0.2**–**4.5**	**0.1**–4.9	**0.2**–0.6	**0.1**–1.6	0.8–1.4
April 17	0.1–0.3	0.1–0.3	**0.1**–0.6	0.2–0.6	0.3–1.2	0.5–3.0	0.3–1.3	0.2–1.0	**0.2**–0.6
July 17	0.4–0.8	0.6–0.9	0.4–0.8	0.2–0.4	1.0–2.4	0.4–1.9	0.7–1.1	0.4–0.7	0.4–0.5
November 17	0.1–0.4	0.1–0.4	0.2–0.6	0.0–0.5	0.1–1.3	0.0–1.8	0.3–0.7	**0.1**–0.5	0.5–1.2
Range	0.1–1.7	0.02–1.7	0.1–2.9	0.03–1.8	0.2–4.5	0.1–5.8	0.2–2.9	0.1–2.2	0.2–5.9
Max–min difference	1.6	1.7	2.8	1.8	4.3	5.7	2.7	2.1	5.7

Overall minimal and maximal growth rates for each group are highlighted in bold. The range of minimal and maximal growth rates, as well as the difference between the highest and the lowest specific growth rates from all the experimental data shown in this table, are presented in the last row. PRK, total prokaryotes. Bacterioplankton groups were detected with specific HRP-probes Eub 338-I, -II, -III (Eubacteria), Ros537 (*Rhodobacteraceae*), SAR11-441R (SAR11 clade), Gam42a (Gammaproteobacteria), Alt1413 (*Alteromonadaceae*), NOR5-730 (NOR5/OM60 cluster) and CF319a (Bacteroidetes). AAP: aerobic anoxygenic phototrophs.

^a^Data from Ferrera et al.^4^, ^b^data from Sánchez et al.^5^, ^c^data from Ferrera et al.^82^.

**Figure 3:**
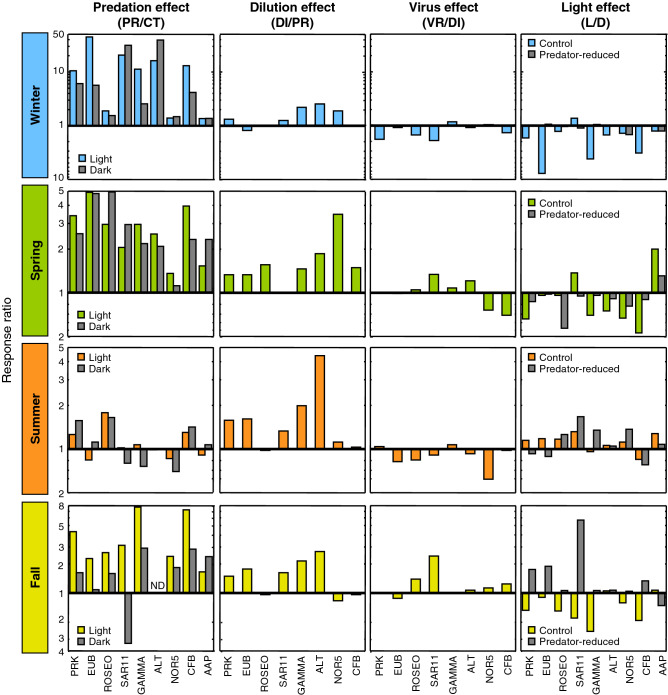
Response ratio of top-down and bottom-up controls calculated from average growth rates of the different groups. The ratio of growth rates between predator-reduced (PR) and control (CT) treatments indicates the effect of grazing. The ratio between diluted (DI) and PR treatments indicates the role of bottom-up control and the ratio between virus-reduced (VR) and DI treatments estimates the effect of viruses. The PAR light effect is given by the ratio between growth rates of light (L) and dark (D) treatments. The 1:1 solid line indicates equal magnitude in both treatments. PRK, total prokaryotes; EUB, Eubacteria; ROSEO, *Rhodobacteraceae*; SAR11, SAR11 clade; GAMMA, Gammaproteobacteria; ALT, *Alteromonadaceae*; NOR5, NOR5/OM60 clade; CFB, Bacteroidetes; AAP, aerobic anoxygenic phototrophs.

**Figure 4:**
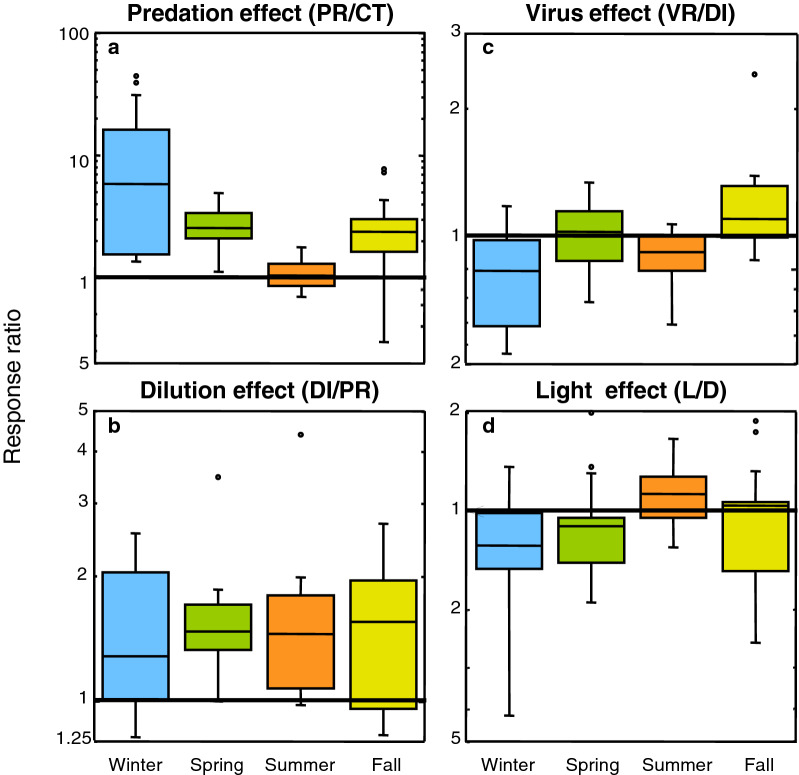
Boxplots showing the distribution and averages of the relative effects of predation (**a**), dilution (**b**), viruses (**c**) and PAR light exposure (**d**) on the growth of all bacterial groups for each season (meaning of the ratios as explained in Fig. 3 legend). From top to bottom, the horizontal lines of the box represent the upper-quartile, median and lower-quartile of the data distributions. Whiskers extending from the top and the bottom of the box represent the largest and the smallest non-outlier value in the data set; outliers were determined as points whose value was either greater or less than the upper quartile plus 1.5 times the interquartile distance. Points plotted separately in the chart represent outliers. The 1:1 solid line indicates that the growth rates have the same magnitude in both treatments.

**Figure 5:**
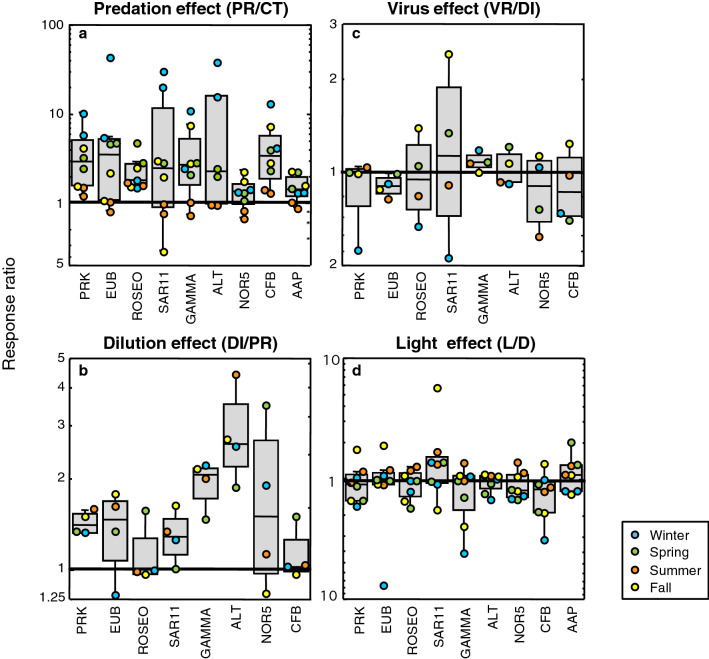
Boxplots showing the relative effects of predation (**a**), dilution (**b**), viruses (**c**) and light exposure (**d**) on the growth for each individual bacterioplankton group. Same graphic explanation as Fig. 4 legend, and same groups as in Fig. 2 legend.

We next compared the growth rates between treatments to investigate what are the key factors controlling the growth of the different bacterioplankton groups. The ratio between predator-reduced vs control treatments growth rates (PR/CT) provides insights on the relative effects of top-down control by grazers (Fig. 3). The PR/CT response ratios indicate that predator removal resulted in increases in the growth rates of all groups studied, especially in winter, followed by spring and fall, while this response was less remarkable in summer (Fig. 4a). Although overall no statistically significant differences in the PR/CT ratio among bacterial groups were found (ANOVA, *p* > 0.05), some variation in the magnitude of this increase can be noted (see Fig. 5a), being less substantial for the NOR5 and AAP groups. In fact, lower growth rates in the predator-reduced treatment compared to the control could be observed in summer and fall for some groups (Figs. 3 and 5a), suggesting that factors other than predation could be controlling the growth rates of these groups at these times of the year.

Comparison of growth rates in the diluted vs predator-reduced treatments (DI/PR) indicates the relative effects of bottom-up control (i.e. resource availability); in this case, while most groups’ growth rates increased when enhancing resource availability, the dilution effect was less important than the predation effect in all seasons but summer, when nutrient limitation was more remarkable. In contrast, the ratio DI/PR was particularly low in winter (Figs. 3 and 4b), when the bacterial community is known to be less limited by nutrients (Table 1); among groups, analysis of variance showed overall significant differences (Fig. 5b, *p* = 0.0091). The dilution effect was more important for the Gammaproteobacteria and its subgroups *Alteromonadaceae* and NOR5 (Fig. 5b), being statistically significant in the case of *Alteromonadaceae* (post hoc Tukey HSD test).

In addition, the virus effect could be determined by comparing the growth rates in the virus-reduced vs the diluted treatment (VR/DI) (Fig. 3). Overall, there was no significant enhancement of growth rates after viral reduction and even some VR/DI ratios lower than 1 could be noticed in all the seasons (Fig. 4c). However, a certain increase in the impact of viruses on the growth rates was observed in the fall compared to the remaining seasons, in concordance with the highest abundance of viruses found in all treatments in this period (Supplementary Fig. S4). In spring, despite the low initial viral abundances (Table 1), there was a small increase in growth rates after viral reduction (Fig. 4c). This could be explained by the comparatively large rise in viral abundance at the end of the treatments detected in this season (Supplementary Fig. S4). When looking at the group-specific viral effect, a small increase of average growth rates for SAR11 and to a lesser extent for Gammaproteobacteria was observed (Fig. 5c).

### Effect of light on growth rates

The light vs dark conditions (L/D) in the controls and predator-reduced treatments were compared to obtain clues on light (i.e. PAR) effect on the growth rates of different bacterial groups. The results of the L/D ratio differed among seasons (Fig. 3). In winter, values were on average equal or higher in the dark than in the light for all groups, with the exception of those for SAR11. In spring, however, besides SAR11, the ratio L/D was also above 1 for the AAP. In summer, interestingly, groups such as the *Rhodobacteraceae*, SAR11, the NOR5 clade, all of them containing photoheterotrophic representatives, as well as the AAP, presented higher growth rates in the light than in the dark. In contrast, the growth rates in the fall were higher in the light only for SAR11 and the Bacteroidetes, both taxa with proteorhodopsin-containing members. Thus, despite no significant differences were found when comparing all growth rates among seasons (ANOVA, *p* > 0,05), the light effect was overall more pronounced in summer than in the other seasons (Fig. 4d), and moreover, SAR11 and, to a certain extent AAP, presented a moderate effect of light on average growth rates (Fig. 5d).

### Mortality rates

Mortality rates, as well as gross growth rates (µ), were calculated in the different experiments from the net growth rates observed in the control, predator-reduced, diluted and virus-reduced treatments using the equations derived from those of Evans et al.^58^ and Pasulka et al.^59^ (Supplementary Table S3). In general, the maximum growth rates determined in this work (Table 3) were very close to the values of µ calculated from these equations for all bacterioplankton groups. Significant differences among seasons in µ values were observed (ANOVA, *p* = 0.0341), being particularly different between winter and fall (Tukey HSD test at *p* < 0.05). The calculated mortality rates confirmed the results obtained from the response ratios discussed above (Supplementary Fig. S5), showing that mortality due to grazers (m_g_) and growth constraints due to resource availability (r_c_) were larger than mortality due to viruses (m_v_). Mortality due to grazers (m_g_) was especially remarkable in winter, whereas competition for resources (r_c_), although being important in all seasons, was particularly low in winter and reached overall higher values in summer. The rates of mortality due to viruses (m_v_) were comparatively low in all seasons, with a certain enhancement in the fall and spring, as also indicated by the VR/DI ratios and viral abundances. In addition, the differences among seasons were significant for the m_g_ values (*p* = 3.61e^−8^) but were not for r_c_ and m_v_ (*p* > 0.05).

## Discussion

Our experiments were designed to get insights into the effect of bottom-up and top-down controls on prokaryotic and bacterial group-specific growth rates in an oligotrophic coastal site from the NW Mediterranean. It must be noted that our estimations suffer from some methodological limitations that are intrinsic to manipulation experiments. Grazing reduction by filtration is likely incomplete since some predators may pass through the filters used (1 µm) and, at the same time, large and particle-attached bacteria, as well as most primary producers, would be excluded, thus decreasing initial bacterial abundances and phytoplankton-derived dissolved organic matter compared to the control, possibly resulting in an underestimation of maximum growth rates. At the same time, some cell lysis and subsequent carbon enrichment could have been caused by filtration during the preparation of the PR, DI and VR treatments. Indeed, the average ratio between the concentration of nutrients at the initial time of the treatments vs the in situ values (Supplementary Table S4) indicates that manipulations lead to a considerable increase in NO_3_^−^ and PO_4_^3−^, particularly in summer. Additionally, besides a reduction in the competition for resources in the diluted treatment, there was also a simultaneous reduction in the predation pressure, since the encounter rates between predators and prey were also reduced. However, dilution with 0.2-µm filtered seawater did not limit the presence of viruses, so that, the virus to prokaryote ratio rose in this treatment. In the virus-reduced treatment, where nutrient availability increased by dilution and a large percentage of grazers and lytic viruses were removed, temperate bacteriophages might still impact bacterial populations. Besides these limitations, another methodological consideration is that CARD-FISH probes may display some coverage and specificity biases^65^. Given the marked seasonality in microbial assemblages in temperate systems^10–14^, it is possible that the overall taxonomic composition of a certain targeted group changes between seasons and consequently so may change the specificity of the probes and therefore, the resulting calculations of abundances. Nevertheless, despite these limitations, our approach provided the first estimation of the relative effects of grazing, resource limitation and viruses on different bacterioplankton groups over a seasonal cycle in an oligotrophic system.

Overall, the growth rates estimated from our data show similar trends to those observed by Teira et al.^3^ in a study of nearly the same bacterial phylogenetic groups carried out in a coastal upwelling system at a similar latitude but with a very dissimilar seasonal pattern (Ría de Vigo). That system is characterized by temperatures ranging only between 13 and 17 °C, and a high annual variability in Chl *a* (0.8–8.4 µg·L^−1^) and nutrient content due to strong upwelling events. In particular, Teira et al^3^ found in dilution (1:10) experiments that SAR11 presented low growth rates, while *Rhodobacteraceae* and Gammaproteobacteria exhibited a higher growth potential, as in the BBMO. However, our results and those from Teira et al.^3^ present differences when comparing the different seasons: while in the BBMO *Rhodobacteraceae*, SAR11, Gammaproteobacteria and Bacteroidetes exhibited the highest growth rates in winter (0.96, 0.93, 3.86 and 1.59 day^−1^ in dilution experiments respectively), in the Ria de Vigo Gammaproteobacteria and Bacteroidetes had maximal growth rates in summer (average summer values of 1.82 and 1.19 day^−1^ respectively), although they concurred in observing higher rates for *Rhodobacteraceae* (1.45 day^−1^) and SAR11 (0.59 day^−1^) in winter. It should nevertheless be noted that their dilution treatment was 1:10 whereas ours was 1:4, a fact that could affect the observed differences as could the temperature range in Blanes Bay as compared to Ría de Vigo. Yokokawa et al.^39^ similarly reported seasonal variability of growth rates of individual bacterial groups in the Delaware Bay also in 1:10 dilution experiments, being again the Gamma- and the Alphaproteobacteria the groups with the highest growth rates and in summer (4.3 and 5.5 day^−1^ respectively), in contrast with what we observed in the BBMO. This study site is much more eutrophic than Blanes Bay, and possibly no nutrient limitation occurs through the year.

Growth rates inform about the life history strategies of the various bacterioplankton groups, so that they allow to distinguish e.g. between those microorganisms that face predators by having a small size and growing very slowly (*k*-strategists) from those that have faster growth rates and are metabolically versatile large bacteria (*r*-strategists). Consistent with these terms and supported by our results, the SAR11 group would clearly be considered a *k*-strategist, as this clade (as a whole) presents the lowest average minimal and maximal growth rates in the BBMO (between 0.0 and 1.8 day^−1^) compared to other bacterioplankton groups (Table 3). These values are in the same order as the bulk bacterioplankton community growth rates, also in agreement with the fact that they are more abundant than any of the other studied groups. Other authors^1,66,67^ also reported in situ slow growth of SAR11, among the lowest compared to other examined groups, similarly low as those of Actinobacteria and Firmicutes^1^, not studied here since these are not abundant phyla in our study site. Strikingly, SAR11 presented the highest increase in growth rate in the fall, particularly when viruses were removed (Fig. 5c). Actually, abundant viruses have been reported to target *Pelagibacter ubique*, the cultured representative of the SAR11 clade^68^, some of the most abundant ones identified and quantified in the BBMO^69–71^. At the other extreme, we found that the *Alteromonadaceae* presented the highest average growth rates in the BBMO (Table 3), which would be in accordance with an *r* strategy. High growth rates concur with the high rRNA:rDNA ratios found for this group^1,66^, and with its opportunistic response to phytoplankton blooms^13^.

Another feature that can be informed by growth rates is the distinction between oligotrophic and copiotrophic ways of life. Kirchman^1^ hypothesized that oligotrophic bacteria should probably have growth rates closer to those of the bulk community rate, while copiotrophs would have maximum rates ≥ 1.0 day^−1^. Our results confirm that SAR11 follows an oligotroph life style, and its growth rates are always close to those of the total community (Table 3), while *Alteromonadaceae* would belong to the copiotrophic way (maximum rate in BBMO experiments of 5.8 day^−1^). Besides, the growth rates of *Alteromonadaceae* presented a wider range of variation between maximum and minimum values (Table 3), consistent with being capable of fast growth bursts under optimal conditions, while switching to lower rates when conditions become less favorable (due to grazer action or lack of nutrients). This observation is further supported by the high increase in *Alteromonadaceae* growth rates observed in the DI treatments, when resource availability was experimentally increased (Fig. 4c). In contrast, SAR11 showed a rather narrow range of growth rates (Table 3). As expected, those bacteria with low maximal growth rates, and thus considered oligotrophs (SAR11 and to a lesser extent Bacteroidetes) were the most abundant groups. Conversely, the less abundant groups showed higher maximal growth rates, those predictable for copiotrophic life strategies (Supplementary Fig. S6). Actually, the relative abundances of the various specific groups in the different treatments, shown in Fig. 1, with SAR11 as the most abundant group in virtually all treatments, clearly indicate that abundance offers a biased view of bacterioplankton growth rates.

Grazing reduction promoted an expected significant increase in prokaryotic growth for all groups, being the response ratio to predator removal stronger than that to nutrient enhancement in all seasons but in summer, when populations seemed to be clearly limited by nutrients. In that season, the response ratio to dilution was consistently higher than to predation removal, particularly for the group *Alteromonadaceae*, in agreement with their copiotrophic life style. Different studies have reported an increase in prokaryotic growth rate caused by the reduction of grazing pressure under oligotrophic conditions^4–6,8,9,72,73^, but also by a decrease in resource competition^4,5,9^. In the BBMO, although the number of heterotrophic nanoflagelates did not largely vary through seasons at the start of the experiments (Table 1), the effect of predation was more important in winter, as confirmed also by the calculated mortality rates. Concurrently, a previous study at the site reported that total grazing activity on bacterioplankton reached its maximum in winter^74^.

In contrast to the identified effect of predators and nutrients, we observed a relatively weak effect of viruses on growth rates in all seasons, in agreement with previous studies at the BBMO^4,75^, which indicated that bacterial mortality caused by viral lysis does not seem to be very relevant in the oligotrophic northwestern Mediterranean. A 2-year study in the same site comparing viruses and protists concluded that, in general, protists were the main cause of mortality, although during some periods, possibly in response to peaks in resource availability, viruses could equal protists as a source of bacterial mortality^7^. In fact, we observed some effect of viruses in the fall; interestingly, this effect coincided with relatively high viral abundances and also with the minimum concentration of heterotrophic nanoflagellate abundance observed in Blanes Bay in the year of the experiments, an observation already noticed for another coastal site^76^. Specifically for SAR11, both grazing and viruses did seem to play an important role in controlling growth (Fig. 5c). Boras et al^7^ also reported that lysogeny was particularly important in summer and winter in Blanes Bay, particularly when nutrients were in the same range as the concentrations reported in this work. A high relevance of lysogeny might imply a minor impact of viruses on growth rates in these two seasons, as we observed.

Regarding the effect of light on the specific growth rates of the different groups, we did observe an interesting trend. In general, values were on average equal or slightly higher in the dark than in the light. However, in spring and particularly in summer, AAP and certain bacterioplankton groups likely containing photoheterotrophic representatives, such as SAR11 and Bacteroidetes, presented higher growth rates under light conditions. These organisms are capable of deriving energy from light, but while it is widely known that some AAP and proteorhodopsin-containing isolates can grow faster under light conditions in laboratory cultures^77–81^, the role that light plays on their growth under natural conditions remains less clear. Recently, evidence that light can directly stimulate the growth rates of natural populations of AAP was presented^82^ and here we extend these observations, by showing that particularly in summer, growth rates of the *Rhodobacteraceae* and the NOR5 clade, both groups containing AAP representatives, were higher in the light. However, when comparing absolute values of growth rates of AAP from this study with previous studies at the BBMO (Table 3), it can be observed that the higher values were reached in previous studies in which viruses were removed^4^ or phosphate was added^82^, likely because besides predators and light, AAP can also be controlled by resource availability and viruses. We also noted that the highest growth rates of this group occurred in winter, when there were more nutrients. However, in this season growth rates were higher in the dark compared to light, both in the control and the PR experiments (Fig. 5d), thus supporting that light did not play a significantly positive effect and instead nutrient availability was the key factor. Direct and indirect evidences had shown that in our study site, summer conditions are clearly favorable for the growth of AAP under light conditions^14,82,83^. Further studies focusing on the interplay between light and other factors such as predation, viruses, nutrients and dissolved organic matter, would be necessary to resolve the reasons for the observed differences.

Proteorhodopsins are also major contributors to the solar energy captured in the surface Mediterranean Sea^84^. For the SAR11 clade, light-enhanced growth was observed in all seasons, although a major effect was evident in the summer and fall, when inorganic nutrients were lower. Higher growth rates of Bacteroidetes, a group that also has members harboring proteorhodopsins, were detected in the light only in the fall. In all these cases, however, the effects of light on the growth rates of different bacterioplankton groups were much lower and not comparable to the effects of top-down and bottom-up regulation. Yet, we observed an enhancement of growth for those clades containing photoheterotrophic representatives, which was also season-dependent.

Overall, our study represents a significant contribution to understand the seasonal variation in the interplay of various major factors controlling bacterial growth. Our observations confirm that both bottom-up and top-down factors interact in controlling the net growth rates of all examined groups. Further, we provide insights into the effects of light on the growth of natural populations of photoheterotrophic bacteria, comparing this effect to that of nutrient availability and grazing, and demonstrate that these effects vary seasonally. The reported gross and net growth rates may thus become valuable pieces of information to be fed into ocean models incorporating bacterial-mediated carbon fluxes. Nonetheless, further experimental studies combined with higher-resolution methodologies, i.e. sequencing methods, are needed to determine the factors that regulate the dominance of individual taxa over time in order to evaluate the possible contribution of these microbes to biogeochemical processes.

## Supplementary information

Supplementary information.
