# Supplementary material for: Seasonal impact of grazing, viral mortality, resource availability and light on the group-specific growth rates of coastal Mediterranean bacterioplankton
Source: Sci Rep. 2020 Nov 13;10:19773. doi: 10.1038/s41598-020-76590-5 (PMC7666142; doi:10.1038/s41598-020-76590-5)
Supplement: Supplementary file 1 — Supplementary information. [file 41598_2020_76590_MOESM1_ESM.pdf]

Supplementary material

Seasonal impact of grazing, viral mortality, resource availability and light on the group-specific growth rates of coastal Mediterranean bacterioplankton

Olga Sánchez[1]*, Isabel Ferrera[2,3]*, Isabel Mabrito[1], Carlota R. Gazulla[1,3], Marta Sebastián[3, 4], Adrià Auladell[3], Carolina Marín-Vindas[3, 5], Clara Cardelús[3], Isabel Sanz-Sáez[3], Massimo C. Pernice[3], Cèlia Marrasé[3], M. Montserrat Sala[3], Josep M. Gasol[3]

[1]Departament de Genètica i Microbiologia. Universitat Autònoma de Barcelona, 08193-Bellaterra, Catalunya, Spain

[2]Centro Oceanográfico de Málaga, Instituto Español de Oceanografía, 29640 Fuengirola, Málaga, Spain

[3]Departament de Biologia Marina i Oceanografia, Institut de Ciències del Mar, ICM-CSIC, E08003-Barcelona, Catalunya, Spain

[4]Instituto de Oceanografía y Cambio Global (IOCAG), Universidad de Las Palmas de Gran Canaria (ULPGC), Telde, Spain

[5]Escuela de Ciencias Biológicas, Universidad Nacional, Heredia, Costa Rica

Running title: *bacterioplankton group-specific growth rates*

Keywords: growth rate, mortality rate, bacterioplankton, seasonality, top-down control, bottom-up control, grazing, resource availability, viruses, light, FISH

*Corresponding authors: Olga Sánchez olga.sanchez@uab.es, tel (+34) 93 586 8022; Isabel Ferrera isabel.ferrera@ieo.es, tel. (+34) 95 247 190

Table S1. Mean ratio between final and initial abundances during the length of incubations (36 h in winter and summer and 48 h in spring and fall) for each individual group in each experiment and treatment. PRK, total prokaryotes as measured by DAPI staining; EUB, Eubacteria; ROSEO, *Rhodobacteraceae*; SAR11, SAR11 clade; GAMMA, Gammaproteobacteria; ALT, *Alteromonadaceae*; NOR5, NOR5/OM60 clade; CFB, Bacteroidetes; AAP, aerobic anoxygenic phototrophic bacteria. CT_L, control under PAR; CT_D, control in the dark; PR_L, predator-reduced treatment under PAR; PR_D, predator-reduced treatment in the dark; DI_L, diluted treatment under PAR; VR_L, virus-reduced treatment under PAR; nd, not determined.

		PRK	EUB	ROSEO	SAR11	GAM	ALT	NOR5	CFB	AAP
**WINTER**	**CT_L**	1.2	1.1	1.9	1.2	1.4	1.5	1.6	1.6	3.7
	**CT_D**	1.1	1.2	2.5	1.2	1.3	1.0	1.4	1.6	3.9
	**PR_L**	2.6	3.2	4.7	2.9	12.2	14.6	1.4	9.9	4.3
	**PR_D**	2.4	2.9	4.6	2.3	10.3	13.9	1.6	9.5	6.3
	**DI_L**	2.2	3.1	4.0	4.4	8.3	9.1	1.5	10.4	nd
	**VR_L**	1.6	1.4	2.7	1.8	7.3	10.2	2.4	3.6	nd
**SPRING**	**CT_L**	1.1	1.0	1.2	1.9	1.8	3.0	1.6	1.4	7.5
	**CT_D**	1.2	1.2	1.3	1.3	2.2	4.3	1.9	1.9	7.2
	**PR_L**	1.5	1.6	2.3	2.3	4.7	12.3	2.3	3.6	6.8
	**PR_D**	1.5	1.9	2.5	2.2	4.8	16.4	2.4	3.8	5.4
	**DI_L**	1.7	1.9	2.9	2.1	9.3	104.0	4.7	6.8	nd
	**VR_L**	1.7	2.0	2.9	2.4	12.0	222.0	3.8	3.9	nd
**SUMMER**	**CT_L**	2.1	2.9	2.7	1.8	5.0	1.5	5.6	2.2	3.3
	**CT_D**	1.8	2.0	2.1	1.3	3.7	1.5	4.7	2.4	2.3
	**PR_L**	1.7	1.8	2.2	1.2	3.0	1.4	2.7	1.8	2.7
	**PR_D**	1.8	2.0	2.2	1.5	2.7	1.4	3.1	2.9	1.5
	**DI_L**	3.3	3.8	3.0	1.7	21.6	22.0	4.8	1.6	nd
	**VR_L**	2.6	2.2	2.1	1.9	16.1	8.1	1.8	2.2	nd
**FALL**	**CT_L**	1.1	1.2	1.3	1.0	1.2	0.5	2.0	1.0	4.0
	**CT_D**	1.4	1.4	1.9	1.2	1.8	0.3	2.8	1.5	2.0
	**PR_L**	2.0	1.7	2.7	1.3	4.3	3.6	4.6	2.3	17.5
	**PR_D**	1.4	1.4	2.4	1.2	4.0	4.1	3.0	1.9	21.9
	**DI_L**	2.0	2.0	2.5	1.5	11.5	16.0	2.7	2.0	nd
	**VR_L**	1.7	2.0	3.2	1.8	8.4	16.8	2.1	1.2	nd

Table S2. PERMANOVA analysis of the relative importance of season, treatment and sampling time covariates against the abundance distribution of the FISH taxa. Df, degrees of freedom; Sum of Sqs, sequential sum of squares; Mean Sqs, Mean Squares; F Model, F value by permutation; R^2, partial R-squared. P-values based on 999 permutations (the lowest P-value possible is obtained).

Source	df	Sums of Sqs	Mean Sqs	F model	R^2	Pr (>F)
**Season**	3	123.54	41.2	14.69	0.22	0.001
**Treatment**	5	114.18	22.8	8.15	0.20	0.001
**Sampling time**	3	96.85	32.2	11.52	0.17	0.001
**Residuals**	84	235.44	2.8		0.41	
**Total**	95	570			1	

Table S3. Mean growth and mortality rates (day^{-1}) ± standard deviation of the different bacterioplankton groups in the different seasonal experiments under PAR. Gross growth rates (μ) and mortality estimates were calculated from the net growth rates observed in the control, predator-reduced, diluted and virus-reduced treatments using the equations described in Methods; m_g is the mortality rate due to grazing, r_c the growth constraints due to resource limitation and m_v the mortality due to viruses. Bacterioplankton groups as detected with DAPI (Prokaryotes) and specific HRP-probes Eub 338-II-III (Eubacteria), Ros537 (*Rhodobacteraceae*), SAR11-441R (SAR11 clade), Gam42a (Gammaproteobacteria), Alt1413 (*Alteromonadaceae*), NOR5-730 (NOR5/OM60 cluster) and CF319a (Bacteroidetes).

Group	Winter (Feb 2017)	Spring (April 2017)	Summer (July 2017)	Fall (Nov 2017)
Prokaryotes				
μ	0.68 ± 0.12	0.31 ± 0.05	1.02 ± 0.19	0.54 ± 0.29
m_g	0.55 ± 0.31	0.13 ± 0.08	0.11 ± 0.15	0.19 ± 0.14
r_c	0.00 ± 0.00	0.02 ± 0.03	0.40 ± 0.06	0.18 ± 0.12
m_v	0.06 ± 0.11	0.11 ± 0.09	0.10 ± 0.09	0.00 ± 0.00
Eubacteria				
μ	0.82 ± 0.09	0.40 ± 0.13	0.84 ± 0.24	0.48 ± 0.08
m_g	0.75 ± 0.06	0.17 ± 0.04	0.02 ± 0.04	0.14 ± 0.12
r_c	0.02 ± 0.04	0.00 ± 0.00	0.05 ± 0.08	0.08 ± 0.13
m_v	0.03 ± 0.05	0.18 ± 0.15	0.00 ± 0.00	0.15 ± 0.13
Rhodobacteraceae				
μ	1.00 ± 0.08	0.40 ± 0.13	0.77 ± 0.16	0.67 ± 0.06
m_g	0.46 ± 0.15	0.17 ± 0.04	0.33 ± 0.14	0.27 ± 0.19
r_c	0.03 ± 0.03	0.00 ± 0.00	0.02 ± 0.03	0.04 ± 0.07
m_v	0.02 ± 0.00	0.18 ± 0.15	0.02 ± 0.00	0.22 ± 0.20
SAR11				
μ	0.79 ± 0.13	0.55 ± 0.06	0.67 ± 047	0.65 ± 0.23
m_g	0.71 ± 0.08	0.22 ± 0.08	0.02 ± 0.03	0.14 ± 0.13
r_c	0.05 ± 0.07	0.10 ± 0.08	0.08 ± 0.07	0.14± 0.18
m_v	0.00 ± 0.00	0.03 ± 0.04	0.01 ± 0.02	0.38 ± 0.17
Gammaproteobacteria				
μ	5.28 ± 1.68	1.55 ± 0.10	2.94 ± 0.81	1.84 ± 0.12
m_g	2.15 ± 1.05	0.64 ± 0.13	0.07 ± 0.07	0.55 ± 0.12
r_c	1.34 ± 2.21	0.57 ± 0.22	1.45 ± 0.33	1.10 ± 0.06
m_v	0.00 ± 0.00	0.07 ± 0.11	0.00 ± 0.00	0.10 ± 0.18
Alteromonadaceae				
μ	5.53 ± 1.35	3.62 ± 0.47	2.28 ± 0.17	2.53 ± 0.12
m_g	2.53 ± 1.14	0.80 ± 0.41	0.00 ± 0.00	1.04 ± 0.27
r_c	0.28 ± 0.37	1.13 ± 0.98	1.98 ± 0.32	1.73 ± 0.10
m_v	2.60 ± 2.28	0.49 ± 0.69	0.00 ± 0.00	0.17 ± 0.21
NOR5/OM60				
μ	0.71 ± 0.15	1.26 ± 0.28	1.09	0.79 ± 0.23
m_g	0.13 ± 0.15	0.16 ± 0.17	0.00	0.39 ± 0.19
r_c	0.14 ± 0.12	0.83 ± 0.38	0.17	0.00 ± 0.00
m_v	0.25 ± 0.44	0.00 ± 0.00	0.00	0.11 ± 0.18
Bacteroidetes				
μ	1.66 ± 0.19	0.91 ± 0.15	0.61 ± 0.06	0.56 ± 0.16
m_g	1.49 ± 0.25	0.56 ± 0.08	0.13 ± 0.07	0.32 ± 0.12
r_c	0.04 ± 0.06	0.19 ± 0.21	0.05 ± 0.07	0.09 ± 0.07
m_v	0.00 ± 0.00	0.00 ± 0.00	0.01 ± 0.02	0.10 ± 0.18

Table S4. Mean ratio between initial and *in situ* nutrient concentrations in each experiment and treatment. Values are the mean of three replicates. Concentrations from fall were not determined. CT_L, control under PAR; CT_D, control in the dark; PR_L, predator-reduced treatment under PAR; PR_D, predator-reduced treatment in the dark; DI_L, diluted treatment under PAR; VR_L, virus-reduced treatment under PAR.

		$N\text{-}NO_3^-$	$N\text{-}NO_2^-$	$N\text{-}NH_4^+$	$Si\text{-}SiO_4^{2-}$	$P\text{-}PO_4^{3-}$
WINTER	CT_L	1.04	1.02	0.82	1.19	2.43
	CT_D	1.04	1.02	0.82	1.19	2.43
	PR_L	2.15	1.02	2.59	1.16	2.40
	PR_D	2.07	1.02	2.59	1.16	2.40
	DI_L	1.14	0.99	0.87	1.12	1.59
	VR_L	1.24	1.01	0.95	1.16	1.37
SPRING	CT_L	1.07	0.85	0.36	1.16	0.79
	CT_D	1.07	0.85	0.36	1.16	0.79
	PR_L	1.18	1.31	0.28	1.25	1.25
	PR_D	1.18	1.31	0.28	1.25	1.25
	DI_L	1.08	0.82	0.31	1.09	0.93
	VR_L	2.27	0.81	0.45	1.41	1.54
SUMMER	CT_L	3.29	1.17	0.94	0.87	2.93
	CT_D	3.29	1.17	0.94	0.87	2.93
	PR_L	3.62	1.69	0.96	0.96	3.67
	PR_D	3.62	1.69	0.96	0.96	3.67
	DI_L	4.68	1.25	0.79	0.79	3.33
	VR_L	8.79	1.28	1.83	0.88	4.07

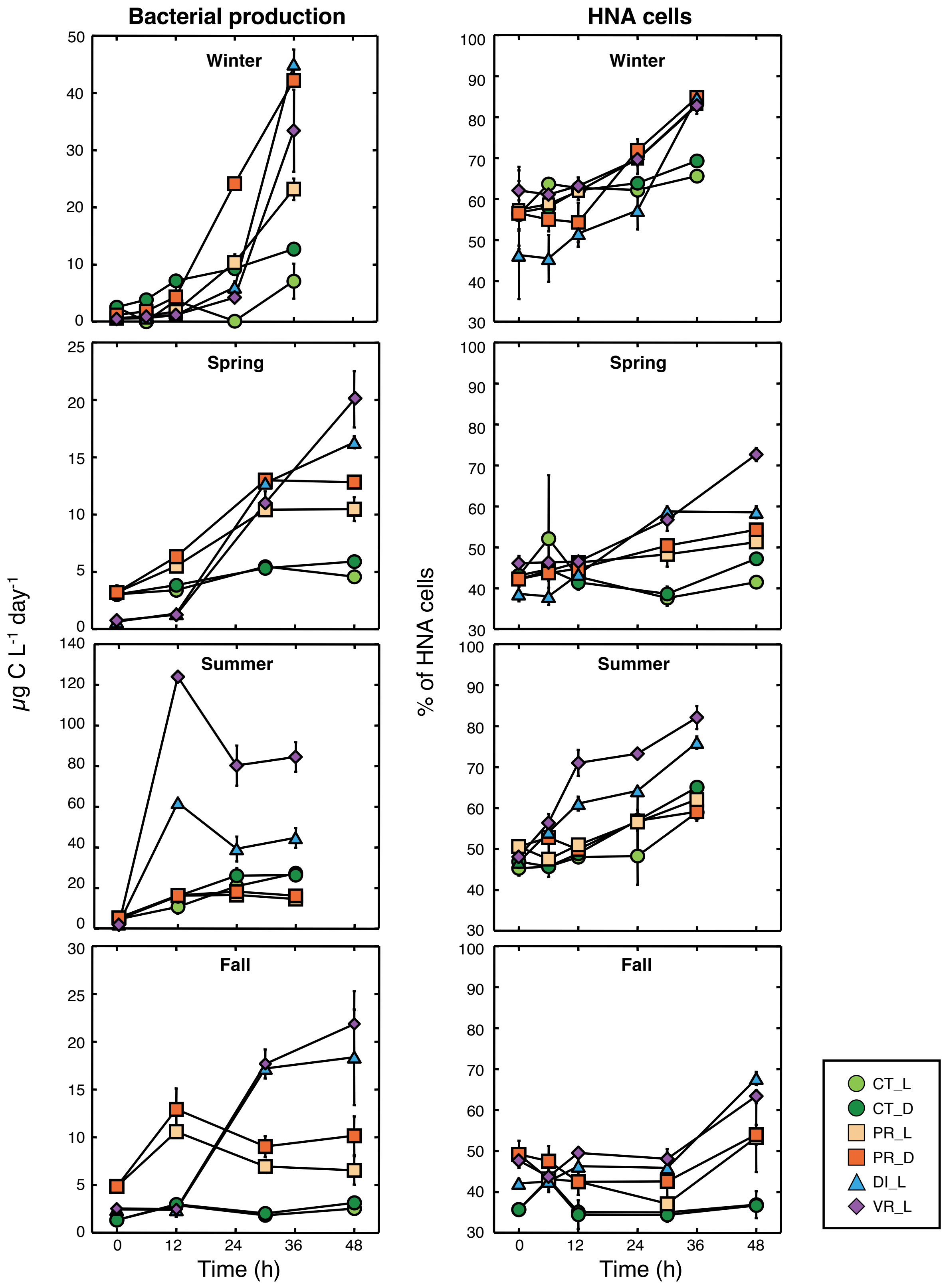

Fig. S1. Left panel: Bacterial heterotrophic production measured as leucine incorporation rates during the incubations for each treatment and experiment. Note the different vertical scales in each experiment. Right panel: Percentage of High Nucleic Acid content (HNA) cells for each treatment and experiment. Error bars represent the standard deviation for three replicated incubations. CT_L, control under PAR; CT_D, control in the dark; PR_L, predator-reduced treatment under PAR, PR_D, Predator-reduced treatment in the dark; DI_L, diluted treatment under PAR; VR_L, virus-reduced treatment under PAR.

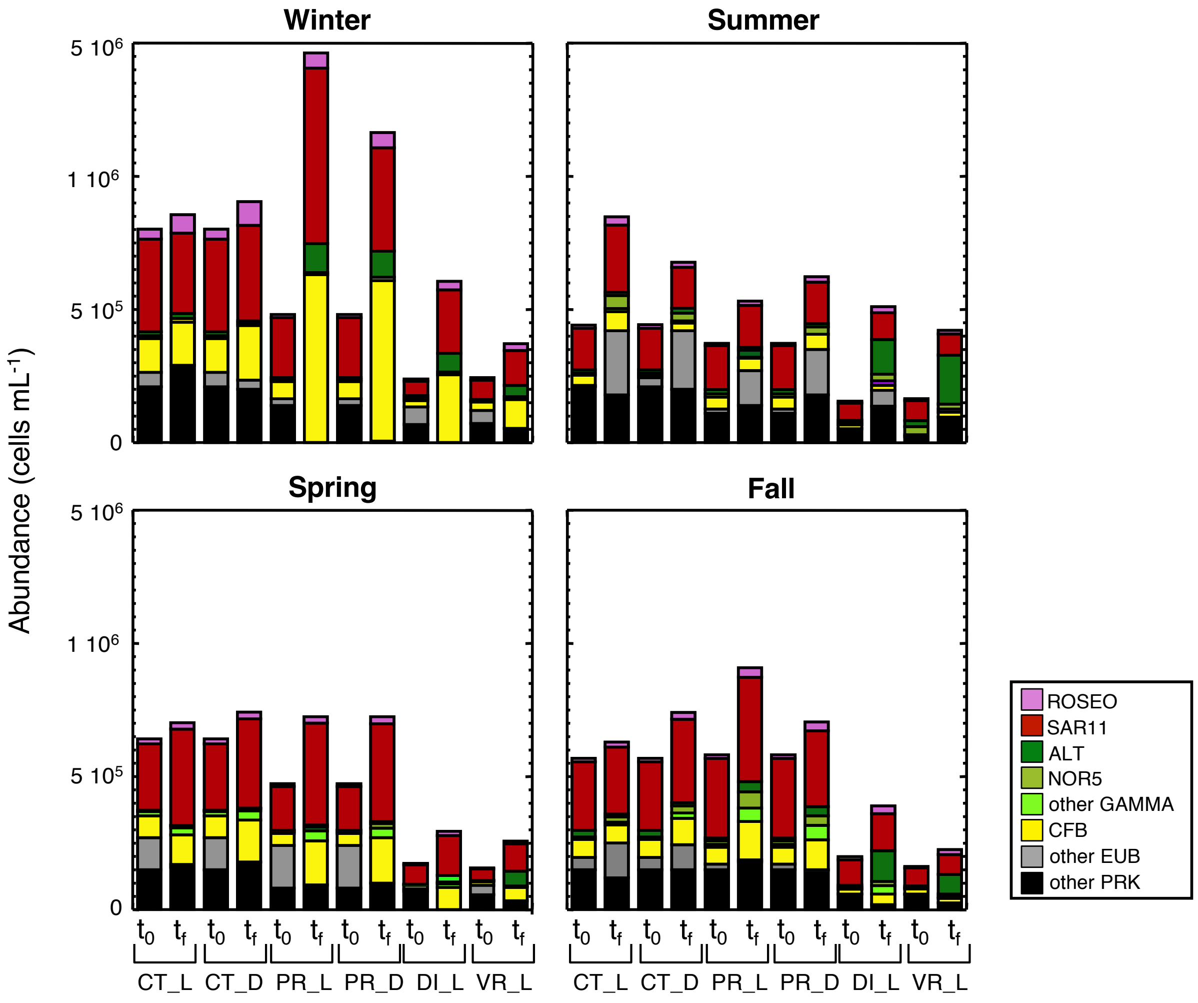

Fig. S2. Stack columns showing the mean abundance of the different phylogenetic groups in each season at the beginning (t_0) and at the end (t_f) of the different treatments (36 h in winter and summer and 48 h in spring and fall). CT_L, control under PAR; CT_D, control in the dark; PR_L, predator-reduced treatment under PAR; PR_D, predator-reduced treatment in the dark; DI_L, diluted treatment under PAR; VR_L, virus-reduced treatment under PAR. ROSEO, *Rhodobacteraceae*; SAR11, SAR11 clade; ALT, *Alteromonadaceae*; NOR5, NOR5/OM60 clade; GAMMA, Gammaproteobacteria; CFB, Bacteroidetes; EUB, Eubacteria; PRK, total prokaryotes (as measured by DAPI staining).

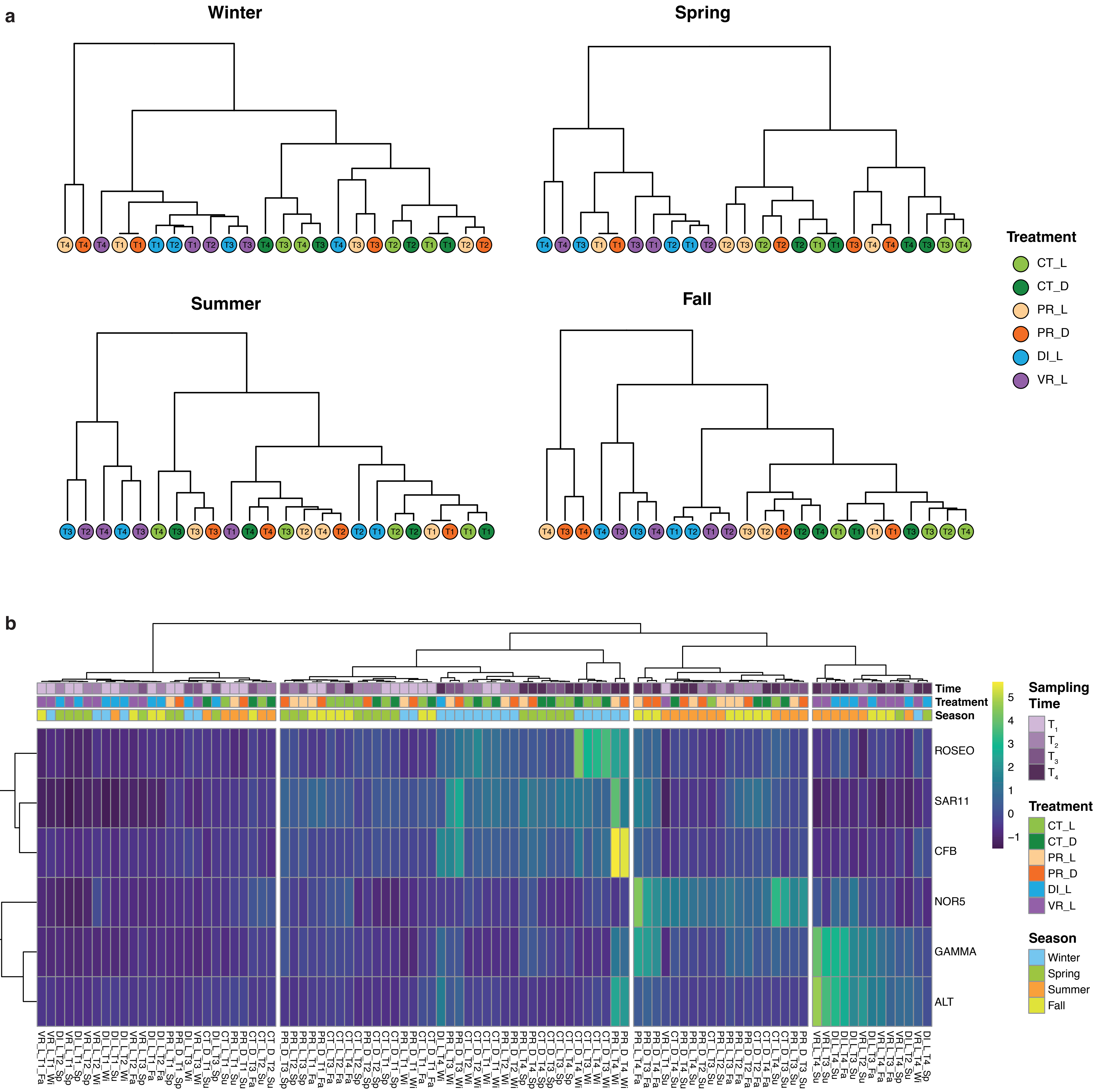

Fig. S3. (a) Hierarchical clustering (Ward's method) based on scaled cell abundances of the group-specific taxa quantified by CARD-FISH in each experiment. Clustering reflects how similar samples are from each other based on their Euclidean distances. (b) Heatmap of all samples together showing the hierarchical clustering by samples (columns) and by taxa (rows). ROSEO, *Rhodobacteraceae*; SAR11, SAR11 clade; CFB, Bacteroidetes; NOR5, NOR5/OM60; GAMMA, Gammaproteobacteria; ALT, *Alteromonadaceae*.

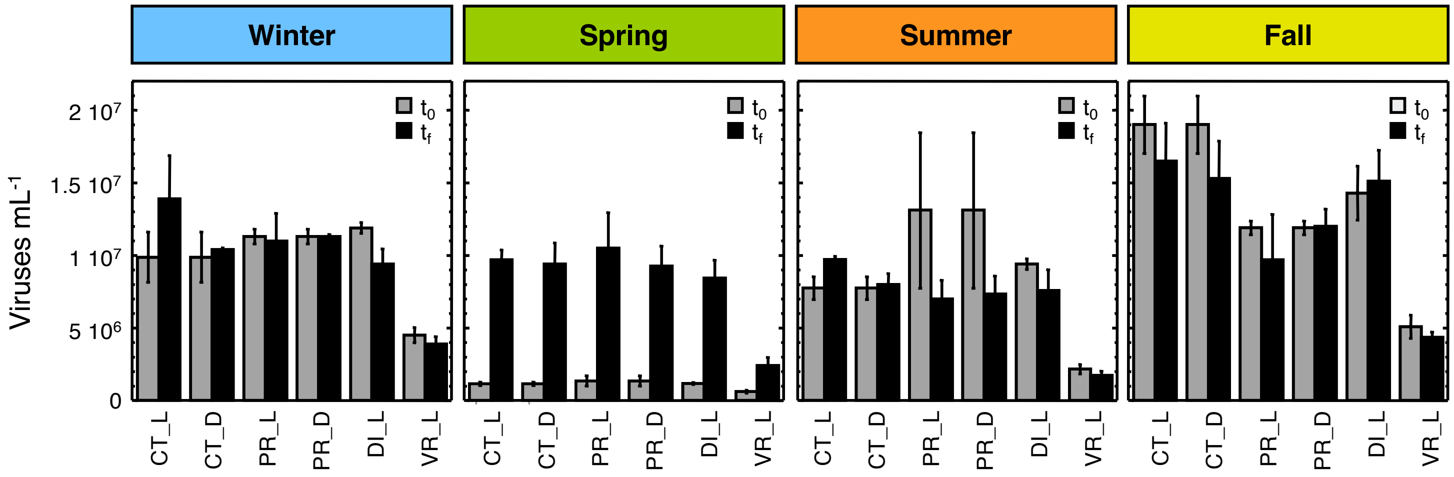

Fig. S4. Mean abundance of viruses in each season at the beginning (t_0) and at the end (t_f) of the different treatments (36 h in winter and summer and 48 h in spring and fall). Error bars represent the standard deviations for three replicated incubations. CT_L, control under PAR; CT_D, control in the dark; PR_L, predator-reduced treatment under PAR; PR_D, predator-reduced treatment in the dark; DI_L, diluted treatment under PAR; VR_L, virus-reduced treatment under PAR.

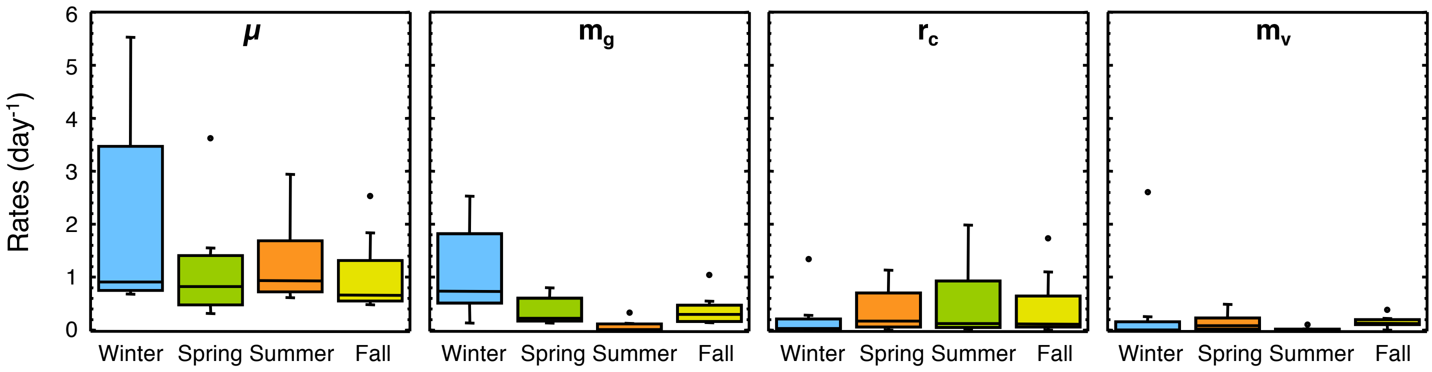

Fig. S5. Boxplots showing the mean values of gross growth (μ) and mortality rates for each individual group due to grazing (m_g) and viruses (m_v), as well as constraints on growth rate due to resource limitation (r_c) in the different seasonal experiments under PAR. From top to bottom, the horizontal lines of the box represent the upper-quartile, median and lower-quartile of the data distributions. Whiskers extending from the top and the bottom of the box represent the largest and the smallest non-outlier value in the data set; outliers were determined as points whose value was either greater or less than the upper quartile plus 1.5 times the interquartile distance. Points plotted separately in the chart represent outliers.

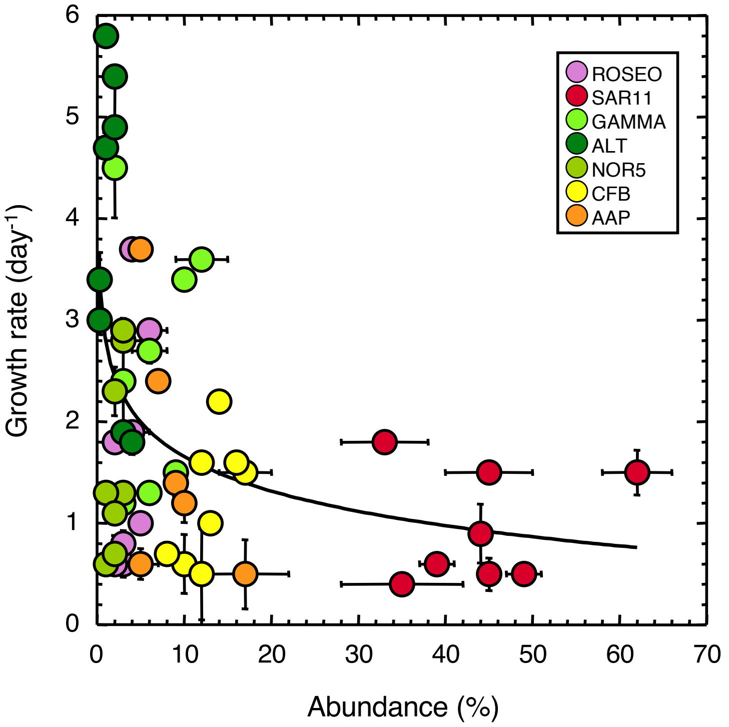

Fig. S6. Relationship between the maximal growth rate found in the BBMO for different bacterioplankton groups and their respective abundances. Error bars represent the standard deviations. ROSEO, *Rhodobacteraceae*; SAR11, SAR11 clade; GAMMA, Gammaproteobacteria; ALT, *Alteromonadaceae*; NOR5, NOR5/OM60 clade; CFB, Bacteroidetes; AAP, aerobic anoxygenic phototrophic bacteria.
